# Pediatric Climbers Experience More Upper Extremity Injuries, Geriatric Climbers More Lower Extremity Injuries, and Increased Shoulder Trauma Associated With Male Individuals in an Emergency Department Analysis of Rock Climbing–Associated Musculoskeletal Injuries

**DOI:** 10.1002/ars2.70015

**Published:** 2026-04-28

**Authors:** Titan Z. Alexio, Philip M. Lee, Nathan N. Kim, Keinan B. Agonias, Kyle K. Obana, Andrew J. Luzzi, Jennifer M. Weiss

**Affiliations:** ^1^ John A. Burns School of Medicine Honolulu Hawaii U.S.A.; ^2^ Division of Orthopedic Surgery University of Hawaii Honolulu Hawaii U.S.A.; ^3^ Department of Orthopedic Surgery Irving Medical Center/NewYork‐Presbyterian Hospital Columbia University New York New York U.S.A.; ^4^ Division of Orthopedic Surgery Shriners Children Medical Center Honolulu Hawaii U.S.A.

## Abstract

**Purpose:**

To analyze epidemiologic trends, injury prevalence, and mechanisms of injury associated with rock climbing.

**Methods:**

The National Electronic Injury Surveillance System, a public emergency department database, was queried for rock climbing–related injuries from 2014 to 2023. Data extracted included demographics, anatomic region injured, diagnosis, mechanism of injury, and disposition. Patients were stratified into 4 age groups: pediatric (≲18 years), young adult (19‐39 years), middle‐aged adult (40‐64 years), and geriatric (≳65 years). Mechanisms of injury were categorized and analyzed by fall height where applicable.

**Results:**

A total of 1346 cases of rock climbing–related injuries were identified after exclusion criteria. Fractures (30.2%) and sprains/strains (20.2%) were the most common diagnoses. The majority of injuries resulted from falls (61.2%), with fracture incidence increasing with fall height. Pediatric and young adult climbers sustained more upper extremity injuries, while geriatric climbers had a higher proportion of lower extremity injuries.

**Conclusions:**

In this study, we found that younger climbers experienced more upper extremity injuries, while older adults more commonly sustained lower extremity injuries. In addition, male rock climbers experienced higher proportions of shoulder injuries compared with female rock climbers. These findings illustrate the potential need for age‐ and sex‐specific injury prevention strategies to guide safe participation in this growing sport.

**Level of Evidence:**

Level IV, retrospective epidemiological case series.

## Graphical abstract

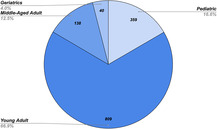

The popularity of rock climbing has grown rapidly over the past several years, particularly following its debut as an official sport in the 2020 Tokyo Olympic Games.^1^ Since 2019, rock climbing has been growing in popularity within the United States, with nearly 6 million climbers engaging in the sport recreationally and competitively.^2^ As participation increases, there is an increased burden of injury, especially given the physically demanding and high‐impact nature of the sport. One study estimated that more than $20 million per year is spent on emergency department visits alone for climbing‐related injuries, highlighting the substantial financial burden these injuries place on the health care system.^3^

Previous studies have documented a wide range of injuries associated with rock climbing.^4^, ^5^ Upper extremity injuries are frequently reported in climbers, with tendon strains and shoulder instability resulting from overuse injuries and acute trauma from fractures and dislocations.^4^, ^6^ Additionally, lower extremity injuries represent a substantial portion of climbing‐related trauma, with Lum et al. reporting 19.9% of all injuries affecting the foot or ankle, often due to falls or improper landings.^7^ When stratifying by age, several studies reported pediatric climbers were more likely to sustain upper extremity injuries, particularly to the forearm and elbow, whereas older adults experienced a higher proportion of lower extremity trauma including injuries to the knee, lower leg, ankle, and foot.^8^, ^9^, ^10^

Although previous literature has attempted to describe overall trends of rock climbing–associated injuries, these studies were conducted prior to 2020 and may not reflect the contemporary injury trends following rock climbing's inclusion in the Olympics. Furthermore, several prior studies included other forms of climbing, such as mountaineering or hiking, which may limit external validity to rock climbing. The purpose of this study was to analyze epidemiologic trends, injury prevalence, and mechanisms of injury (MOIs) associated with rock climbing. We hypothesize that pediatric climbers will have higher injury rates, especially to the upper extremity, and geriatric climbers will have increased lower extremity injuries.

## METHODS

### Study Design

This study utilized the National Electronic Injury Surveillance System (NEISS) database from January 1, 2014, to December 31, 2023, for all rock climbing–related injuries, with relevant cases identified using NEISS product codes and keyword filters of injury narratives. The NEISS dataset is a publicly available dataset provided by the Consumer Product Safety Commission and includes data from more than 5000 emergency departments and 100 hospitals across the United States. Variables including patient demographics, date of presentation, anatomic region injured, and final diagnosis are provided within the dataset. Based on hospital size and geographic location region, injury cases are assigned a statistical weight, and previously published literature has validated calculation of National Estimates (NEs) by multiplying the associated statistical weight by their raw data point.^11^, ^12^

Each NEISS record contains a 400‐character narrative text field provided by an emergency department clinician, providing information on the sequence of events and MOI. The first 2 authors (T.A., P.L.) reviewed all narratives to ensure the injury primarily occurred while rock climbing, as well as assigned patient cases an MOI. In the scenario of disagreement on narrative interpretation, J.W. would be consulted. Cases were included in the analysis if the injury occurred while rock climbing and were documented in the NEISS database after January 1, 2014. Cases were excluded if narratives described non–rock climbing–related injuries (e.g., injured while walking to a rock‐climbing gym), secondary injuries associated with another primary mechanism (e.g., initial injury while playing volleyball, exacerbated with rock climbing), or a primary rock‐climbing injury exacerbated with another mechanism (e.g., injured while rock climbing, exacerbated at baseball practice).

### Outcomes of Interest

Outcomes of interest included the anatomic region injured, final diagnosis, and mechanism of injury. These outcomes were stratified by age into 4 groups, including pediatric patients (≤18 years), young adults (19‐39), middle‐aged adults (40‐64), and geriatric patients (≥65 years). MOI was categorized as falls, impact with terrain, equipment failure, exertional cardiovascular event, noncontact movements, and overhead arm movements. Falls were further stratified by height into 4 categories: <10 feet, 10‐20 feet, >20 feet, and unspecified height. If narratives provided insufficient detail or the exact mechanism was unclear, they were classified as “unspecified” (e.g., “injured hand while rock climbing”).

### Statistical Analysis

Statistical analyses were performed using IBM SPSS software (version 29.0; IBM, Armonk, NY). Based on each hospital's capacity, the NEISS dataset provides a statistical weight for each patient case that can be used to calculate an accurate national estimate to represent national trends.^12^ NEs were then calculated by multiplying the associated statistical weight of the reporting hospital by each queried raw data point. Trends in NE for overall injuries, injury diagnoses, and mechanisms of injury were assessed using linear regressions. Similar analyses were conducted to evaluate trends in age groups, sex, and the year 2020 to account for the effects of the pandemic. Chi‐squared analyses were used to analyze categorical variables. The Holm *P* value adjustment method was used in post hoc analysis. Statistical significance was set at *P* < .05.

## RESULTS

### Patient Demographics

**FIGURE 1:**
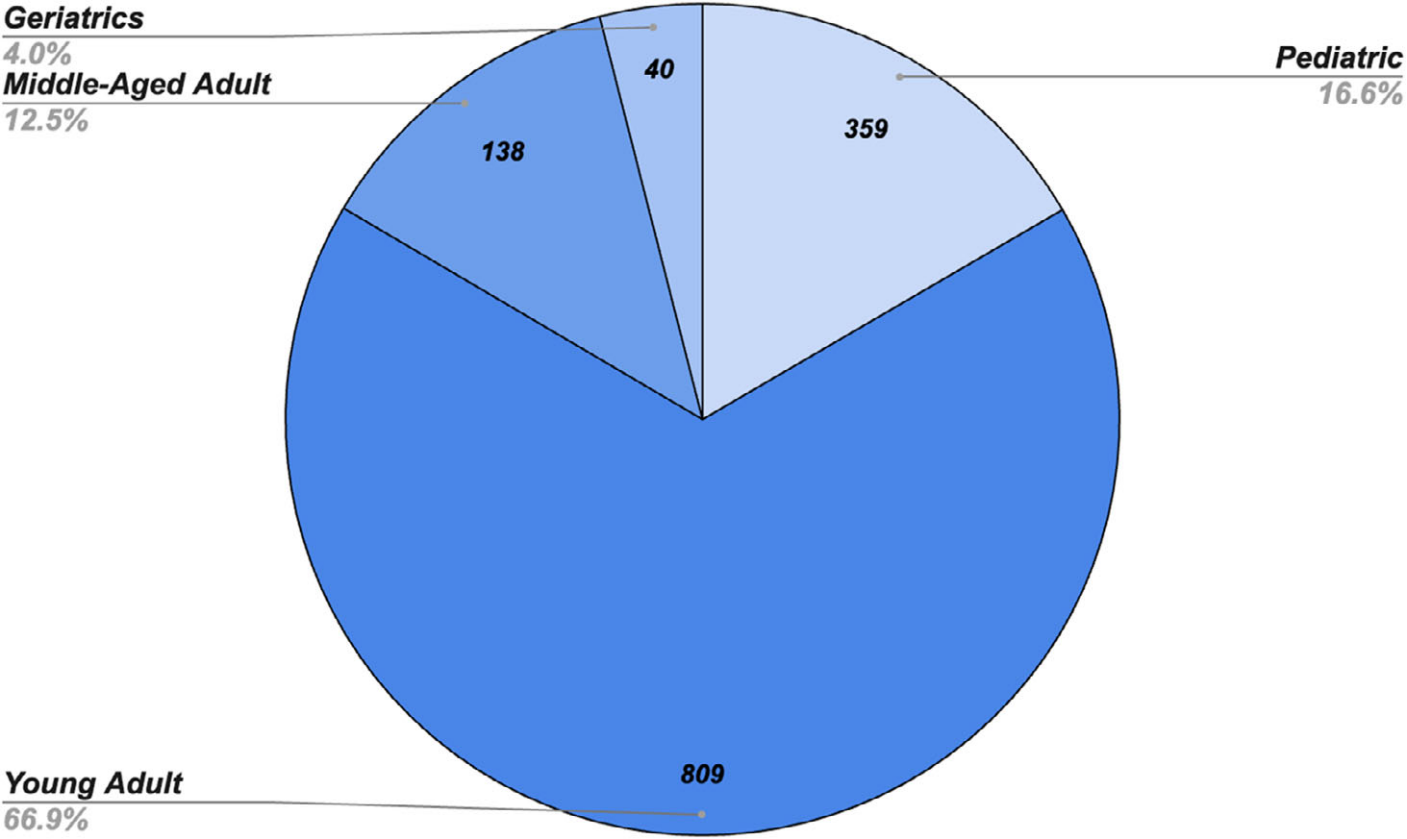
Distribution of rock climbing–associated injuries among different age groups.

There were 1457 (NE = 52,339) reported rock‐climbing injuries from 2014 to 2023. A total of 1346 rock‐climbing injuries met the inclusion criteria and were included in this study. The mean patient age was 27.97 ± 13.59 years, with 359 (NE = 7999, 24.6%) pediatric patients, 809 (NE = 32,270, 55.5%) young adults, 138 (NE = 6050, 9.5%) middle‐aged adults, and 40 (NE = 1916, 2.7%) geriatric patients. Patients were primarily male (56.1%) and non‐Hispanic (38.6%). There were 782 (NE = 31,981, 58.1%) White patients and 91 (NE = 2630, 6.8%) Asian patients. Additionally, 1139 (NE = 41,125, 84.6%) patients were discharged after initial evaluation. Figure 1 illustrates the distribution of rock climbing–associated injuries among different age groups.

### Injury Trends

Among all age groups, the most common anatomic region injured were 278 (NE = 9235, 20.7%) ankles, 120 (NE = 4492, 8.9%) internal organ injuries, and 99 (NE = 4799, 7.4%) upper trunk injuries. There were 375 (NE = 12,304, 27.9%) patients with polytraumatic injuries and 92 (NE = 3266, 6.8%) that were described as other/not stated. Additional distribution of all anatomical locations injured can be found in Table 1.

**TABLE 1:** National Estimates and Percent Distribution of Anatomic Region Within All Climbers

Anatomic Region	NEISS Count	NEISS National Estimate	Percentage, %
Ankle	375	12,304	27.9
Head	120	4492	8.9
Shoulder (including clavicle, collar)	99	4799	7.4
Trunk, upper (not including shoulders)	94	3631	7.0
Trunk, lower	92	3266	6.8
Leg, lower (not including knee or ankle)	84	3397	6.2
Foot	84	3193	6.2
Elbow	84	2413	6.2
Arm, lower (not including elbow or wrist)	54	1299	4.0
Other	54	1900	4
Finger	48	1715	3.6
Hand	40	2020	3.0
Wrist	38	1400	2.8
Face (including eyelid, eye area, and nose)	28	954	2.1
Arm, upper	22	583	1.6
Neck	17	613	1.3
Leg, upper	13	257	1.0

NEISS, National Electronic Injury Surveillance System.

The most common final diagnoses were fractures (n = 406, NE = 13,701, 30.2%), (NE = 9691, 20.2%) sprains/strains (n = 272, NE = 9691, 20.2%), and contusions/abrasions (n = 119, NE = 4340, 8.8%). 241 (NE = 8485, 17.9%) of all final diagnoses were reported as “other/not stated.” All rock climbing–associated final diagnoses are listed in Table 2.

**TABLE 2:** National Estimates and Percent Distribution of Final Diagnoses for All Climbers

Diagnosis	NEISS Count	NEISS National Estimate	Percentage, %
Fracture	406	13,701	30.2
Strain or sprain	272	9691	20.2
Other/Not stated	267	9294	19.7
Contusion, abrasion	119	4340	8.8
Dislocation	89	3534	6.6
Laceration	81	3839	6
Internal organ injury	72	2207	5.3
Concussion	26	1116	1.9
Nerve damage	8	311	0.6
Hematoma	6	201	0.4

NEISS, National Electronic Injury Surveillance System.

The most common MOI included 824 impacts with the floor (NE = 27,237, 61.2%), 183 (NE = 8050, 13.6%) impacts with the terrain, and 62 (NE = 2645, 4.6%) noncontact movements. There were 148 (NE = 5118, 11.0%) unspecified MOI. All MOIs are listed in Table 3.

**TABLE 3:** National Estimates and Percent Distribution of Mechanism of Injury for All Climbers

Mechanism of Injury	NEISS Count	NEISS National Estimate	Percentage, %
Falls	824	27,237	61.2
Impact with terrain	183	8050	13.6
Unspecified	148	5118	11.0
Noncontact movement	62	2645	4.6
Overhead arm movement	57	2354	4.2
Equipment malfunction	37	1504	2.7
Exertional cardiovascular event	28	1092	2.1

NEISS, National Electronic Injury Surveillance System.

### Most Common Diagnoses of the Top 3 Anatomic Regions Injured

Of all ankle injuries, the most common final diagnoses were 137 (NE = 4533, 49.1%) sprains/strains, 105 (NE = 3573, 38.7%) fractures, and 6 (NE = 199, 2.2%) dislocations. Of all head injuries, the most common final diagnoses were 64 (NE = 1975, 44.0%) internal organ damage, 26 (NE = 1116, 24.8%) concussions, and 16 (NE = 699, 15.6%) lacerations. Of all shoulder injuries, the most common final diagnoses were 42 (NE = 2061, 43.0%), dislocations, 25 (NE = 1076, 22.4%) sprains/strains, and 10 (NE = 618, 12.9%) fractures.

### General Trends Stratified by Age

#### Pediatrics

The most commonly injured anatomic locations were 52 (NE = 1293, 14.5%) ankles, 37 (NE = 744, 10.3%) elbows, and 34 (NE = 594, 9.5%) lower arms. The most common final diagnoses were 112 (NE = 2121, 31.2%) fractures, 72 (NE = 2005, 20.1%) sprains/strains, and 45 (NE = 910, 12.5%) contusions/abrasions. The most common MOIs were 237 (NE = 4645, 66.0%) impacts with the floor, 44 (NE = 1432, 12.3%) impacts with terrain, and 20 (NE = 641, 5.6%) noncontact movements.

#### Young Adults

The most commonly injured anatomic locations were 198 (NE = 6660, 24.5%) ankles, 68 (NE = 3336, 8.4%) shoulders, and 66 (NE = 3037, 8.2%) heads. The most common final diagnoses were 231 (NE = 8717, 28.6%) fractures, 179 (NE = 6504, 22.1%) sprains/strains, and 149 (NE = 5558, 18.4%) contusions/abrasions. The most common MOIs were 485 (NE = 18,356, 60.0%) impacts with the floor, 110 (NE = 5280, 13.6%) impacts with terrain, and 44 (NE = 1863, 5.4%) overhead arm movements.

#### Middle‐Aged Adults

The most commonly injured anatomic locations were 26 (NE = 1192, 18.8%) ankles, 18 (NE = 787, 13.0%) upper trunks, and 15 (NE = 526, 10.9%) lower legs. The most common final diagnoses were 52 (NE = 2207, 37.7%) fractures, 16 (NE = 863, 11.6%) sprains/strains, and 12 (NE = 493, 8.7%) contusions/abrasions. The most common MOIs were 78 (NE = 3191, 56.5%) impacts with the floor, 23 (NE = 996, 16.7%) impacts with terrain, and 6 (NE = 286, 4.3%) exertional cardiovascular events.

#### Geriatrics

The most commonly injured anatomic locations were 6 (NE = 211, 15.0%) knees, 6 (NE = 206, 15.0%) heads, and 5 (NE = 258, 13.5%) upper trunks. The most common final diagnoses were 11 (NE = 657, 34.3%) fractures, 8 (NE = 236, 20.0%) internal organ damage, and 5 (NE = 319, 12.5%) sprains/strains. The most common MOI were 24 (NE = 1045, 60.0%) impacts with the floor, 6 (NE = 341, 15.0%) impacts with terrain, and 3 (NE = 107, 7.5%) noncontact movements.

#### Post Hoc Analysis Between Age Groups

Post hoc analysis revealed the pediatric population had the greatest proportion of contusions/abrasions (11.4%, *P* = .035) and sprains/strains (25.1%, *P *= .022), whereas young adults had the greatest proportion of dislocations (9.1%, *P* < .001). Regarding the most common anatomic region injured, pediatric patients had the greatest proportion of elbow (9.3%, *P* < .001), lower arm (7.4%, *P* < .001), and wrist (6.1%, *P* = .045) injuries compared with all other age groups. Geriatric patients had the greatest proportion of lower leg (12.8%, *P* = .046) injuries compared with all other age groups. All corresponding *P* values can be found in Tables 4, 5, 6.

**TABLE 4:** National Estimates and Percent Distribution of Body Region Within Each Respective Rock‐Climbing Age Group

**Body Region**	**Pediatrics**	**20‐39 Years**	**40‐59 Years**	**60+ Years**	***P*** **Value**	**Total**
Shoulder (including clavicle, collarbone)	464 (5.8%)	3336 (10.3%)	685 (11.3%)	314 (16.4%)	.060	4799
Trunk, upper (not including shoulders)	221 (2.8%)	2365 (7.3%)	787 (13.0%)	258 (13.5%)	.002	3631
Elbow	744 (9.3%)	1433 (4.4%)	180 (3.0%)	56 (2.9%)	<.001	2413
Arm, lower (not including elbow or wrist)	594 (7.4%)	594 (1.8%)	95 (1.6%)	16 (0.8%)	<.001	1299
Wrist	486 (6.1%)	769 (2.4%)	145 (2.4%)	‐	.045	1400
Knee	560 (7.0%)	2066 (6.4%)	231 (3.8%)	211 (11.0%)	.206	3068
Leg, lower (not including knee or ankle)	398 (5.0%)	2228 (6.9%)	526 (8.7%)	245 (12.8%)	.046	3397
Ankle	1293 (16.2%)	6660 (20.6%)	1192 (19.7%)	90 (4.7%)	<.001	9235
Head	819 (10.2%)	3037 (9.4%)	431 (7.1%)	206 (10.7%)	.168	4493
Face (including eyelid, eye area, and nose)	186 (2.3%)	661 (2.0%)	33 (0.6%)	74 (3.8%)	.168	954
Trunk, lower	560 (7.0%)	2118 (6.6%)	369 (6.1%)	219 (11.4%)	.168	3266
Arm, upper	157 (2.0%)	312 (1.0%)	114 (1.9%)	‐	.305	583
Leg, upper	11 (0.1%)	216 (0.7%)	‐	‐	.674	227
Hand	214 (2.7%)	1598 (5.0%)	134 (2.2%)	74 (3.9%)	.363	2020
Foot	350 (4.4%)	2295 (7.1%)	533 (8.8%)	15 (0.8%)	.257	3193
All parts of body (more than 50% of body)	37 (0.5%)	247 (0.8%)	163 (2.7%)	‐	.060	447
Finger	512 (6.4%)	1039 (3.2%)	164 (2.7%)	‐	.524	1715
Other: not recorded, ear, toe, mouth, eyeball, pubic region, neck	396 (5.0%)	1296 (6.3%)	239 (3.9%)	139 (7.2%)	.517	2070

**TABLE 5:** National Estimates and Percent Distribution of Final Diagnosis Within Each Respective Rock‐Climbing Age Group

**Diagnosis**	**Pediatrics**	**20‐39 Years**	**40‐59 Years**	**60+ Years**	***P*** **Value**	**Total**
Concussions	113 (1.4%)	892 (2.8%)	112 (1.8%)	‐	.473	1117
Contusions, abrasions	910 (11.4%)	2844 (8.8%)	493 (8.1%)	93 (4.9%)	.035	4247
Dislocation	306 (3.8%)	2937 (9.1%)	205 (3.4%)	86 (4.5%)	<.001	3534
Fracture	2121 (26.5%)	8717 (27.0%)	2207 (36.5%)	657 (34.3%)	.171	13,702
Laceration	788 (9.8%)	2691 (8.3%)	277 (4.6%)	83 (4.3%)	.454	3839
Internal organ injury	361 (4.5%)	1290 (4.0%)	320 (5.3%)	236 (12.3%)	<.001	2207
Strain or sprain	2005 (25.1%)	6504 (20.2%)	863 (14.3%)	319 (16.7%)	.022	9691
Other/Not stated	1091 (13.6%)	5558 (17.2%)	1431 (23.7%)	405 (21.1%)	.047	8485

**TABLE 6:** National Estimates and Percent Distribution of MOI Within Each Respective Rock‐Climbing Age Group

**MOI**	**Pediatrics**	**20‐39 Years**	**40‐59 Years**	**60+ Years**	***P*** **Value**	**Total**
Fall	4645 (58.1%)	18,356 (56.9%)	3191 (52.7%)	1045 (54.6%)	.149	27,237
Impact with terrain	1432 (17.9%)	5280 (16.4%)	996 (16.5%)	341 (17.8%)	.643	8049
Exertional cardiovascular event	202 (2.5%)	588 (1.8%)	286 (4.7%)	16 (0.8%)	.222	1092
Noncontact movement	641 (8.0%)	1662 (5.2%)	235 (3.9%)	107 (5.6%)	.331	2645
Overhead arm movement	52 (0.7%)	1863 (5.8%)	274 (4.5%)	165 (8.6%)	.018	2354
Equipment failure	305 (3.8%)	1047 (3.2%)	152 (2.5%)	‐	.640	1504
Unspecified	697 (8.7%)	3328 (10.3%)	852 (14.1%)	241 (12.6%)	.718	4421

MOI, mechanism of injury.

#### Injury Patterns Stratified by Fall Height

**FIGURE 2:**
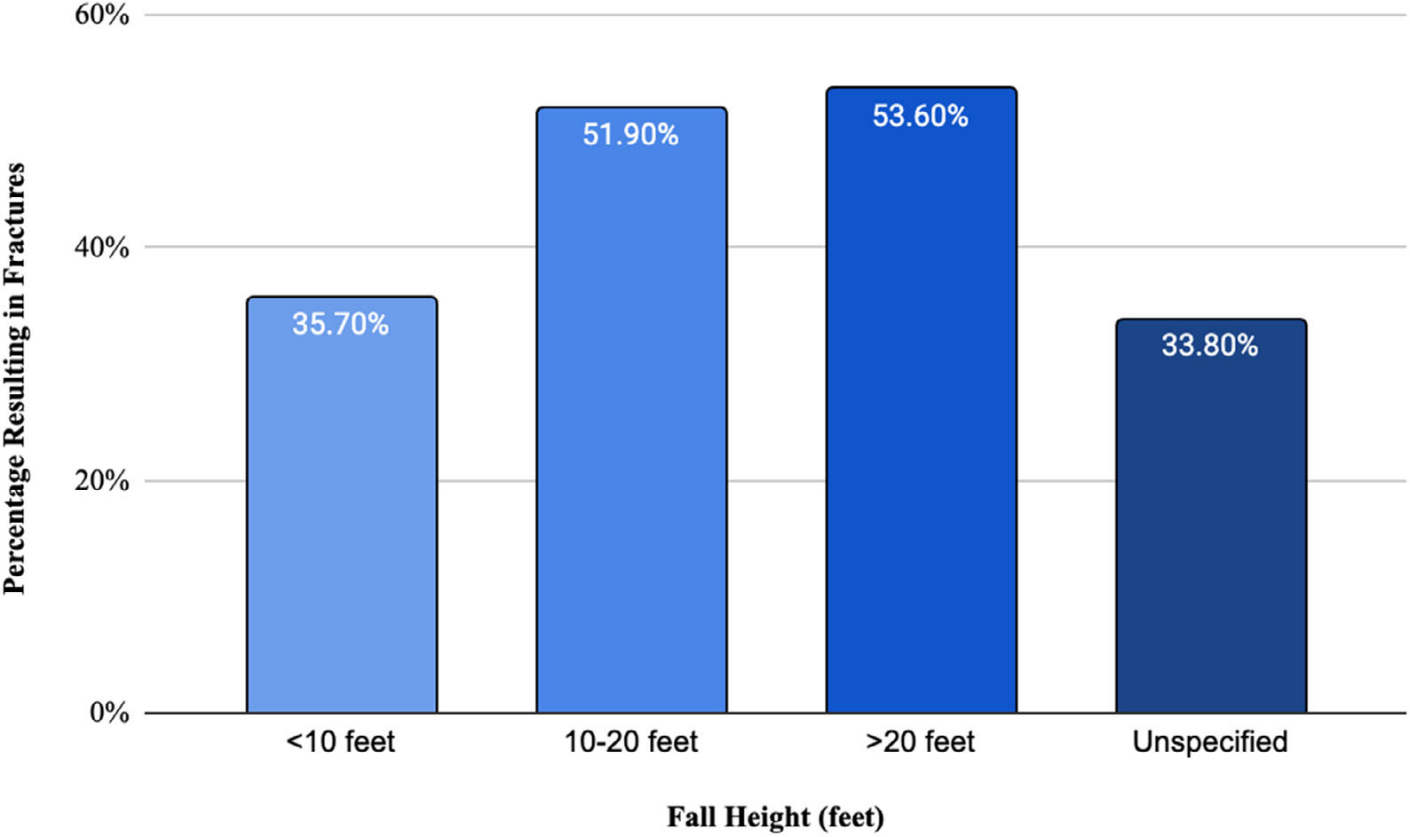
Rates of fractures when stratified by different heights.

Of injuries secondary to an impact with the floor, there were 140 (NE = 4254, 10.4%) falls from <10 feet, 189 (NE = 7146, 14.1%) falls from 10 to 20 ft, 84 (NE = 3403, 6.2%) falls from >20 feet, and 411 (NE = 12,435, 30.5%) falls from an unspecified height. Additionally, of all diagnoses secondary to falls of various heights, there were 50 (NE = 1611, 35.7%) fractures from falls <10 feet, 98 (NE = 3486, 51.9%) fractures from falls 10 to 20 feet, and 45 (NE = 1678, 53.6%) from falls >20 feet. Figure 2 illustrates rates of fractures when stratified by different heights.

#### Injury Patterns Stratified by Sex

Of all male injuries, the most common body regions injured included 119 (NE = 4184, 14.4%) ankles, 75 (NE = 3762, 13.0%) shoulders, and 67 (NE = 2511, 8.6%) heads. The most common final diagnoses included 358 (NE = 8285, 28.6%) fractures, 138 (NE = 5263, 18.1%) sprains/strains, and 57 (NE = 2522, 8.7%) dislocations. The most common MOIs were 446 (NE = 16,134, 55.6%) falls, 110 (NE = 5052, 17.4%) impact with terrain, and 36 (NE = 1425, 4.9%) overhead arm movements.

Of all female injuries, the most common body regions injured included 159 (NE = 5051, 26.3%) ankles, 53 (NE = 1981, 10.3%) heads, and 46 (NE = 1459, 7.6%) knees. The most common final diagnoses included 187 (NE = 5661, 29.5%) fractures, 134 (NE = 4429, 23.1%) sprains/strains, and 55 (NE = 1670, 8.7%) contusions/abrasions. The most common MOIs were 378 (NE = 11,102, 57.8%) falls, 73 (NE = 2998, 15.6%) impact with terrain, and 30 (NE = 1399, 7.3%) noncontact movements.

Post hoc analyses showed that female individuals had a significantly higher proportion of sprains/strains (*P* = .027) and injuries secondary to falls (*P* = .038), whereas male individuals had a significantly higher proportion of shoulder injuries (*P* < .001). There were no significant differences in proportions of fractures (*P* = .193); contusions (*P* = .331); dislocations (*P* = .072); injuries to the wrist (*P* = .103), head (*P* = .513), finger (*P* = .224), knee (*P* = .268), or elbow (*P* = .101); or injuries secondary to impact with terrain (*P* = .136) or overhead movements (*P* = .168).

## DISCUSSION

Fractures (30.2%) and sprains/strains (20.2%) were the most common diagnoses overall. Young adults accounted for the highest proportion of rock climbing–related injuries in our cohort, representing a significant portion of nationwide cases based on national estimates (NE = 32,270, 55.5%). Pediatric and young adult climbers experienced a higher proportion of upper extremity injuries, particularly to the elbow (9.3%) and lower arm (7.4%) in pediatric patients, while older adults showed a greater frequency of lower extremity injuries, including the knee (11.0%), lower leg (12.8%), and lower trunk (11.4%). Additionally, the majority of injuries in our cohort resulted from falls (61.2%). Data analysis revealed that fractures occurred in 53.6% of falls from greater than 20 feet, compared with 35.7% of falls from less than 10 feet.

Ankles were the most frequently injured body region overall (27.9%), with 77% of all ankle injuries secondary to falls. This aligns with the current evidence, with Identeg et al. reporting that foot and ankle sprains accounted for 72% of rock‐climbing injuries, with 84% of all injuries being attributed to falls.^13^ Additionally, fracture incidence was strongly associated with fall height. There is strong evidence showing falls from a greater height correlate with more severe and frequent injuries due to greater kinetic energy from acceleration, with falls from just 8 feet having the potential to result in fractures to the calcaneus, talus, and ankle joint.^12^, ^14^, ^15^ Upon impact with the floor, significant axial loading is placed on the ankle joint, and depending on the positioning of the foot, load can be unevenly distributed, leading to significant strain on the surrounding bone and ligaments. A systematic review by Bellows and Wong evaluated multiple randomized controlled trials among several sports, finding the use of ankle braces led to a 64% reduction in ankle sprains compared with those who did not; therefore, athletes should consider utilizing protective equipment when engaging in rock climbing.^16^

In pediatric climbers, upper extremity injuries were more frequently observed than in other age groups, with the elbow (10.3%) and lower arm (9.5%) being the most commonly injured anatomical regions. This aligns with prior literature showing that younger athletes are particularly vulnerable to upper extremity trauma associated with rock climbing.^17^ Additionally, there is evidence suggesting children may more often exhibit upper extremity motor patterns such as the arm rescue reaction, a reflexive extension of the upper limbs during falls, predisposing trauma to vulnerable joints like the elbow and wrist.^18^ These age‐specific biomechanical factors, coupled with lower coordination during physical activity, likely explain the elevated incidence of upper extremity trauma among young climbers.^16^, ^18^

Geriatric rock climbers had the greatest percentage of lower extremity injuries, with the lower leg (12.8%), knee (11.0%), and lower trunk (11.4%) being the most common sites of injury. Similar to all age groups, falls were responsible for a majority of the injuries and occurred in 60% of geriatric injuries. The large proportion of lower extremity injuries can be explained by the age‐related declines in joint stability and bone loss, similar to middle‐aged adults, but also by sensory reflex decline.^19^, ^20^, ^21^ Lockhart et al. reported that deterioration of lower extremity muscle strength and sensory reflexes in older individuals often prevents them from adequately reacting to a fall, subsequently leading to significant lower extremity trauma.^22^ Age‐specific impairment of joint and bone strength, reflexes, coordination, and processing time help to explain the increased rate of lower extremity injuries in the geriatric population.

This study identifies significant differences in injury patterns among different age groups participating in rock climbing. Given the high risk for injury in the sport, our study provides potential implications to reduce the incidence in novice and experienced climbers. For pediatric patients, protective measures should focus on reducing upper extremity trauma, such as wrist guards or padded landing zones, as well as instruction on proper fall mechanics to minimize fall onto out‐stretched hand‐related injuries. In young adults, who often pursue more dynamic or technical routes, targeted education on technique refinement, controlled movement, and safe belaying practices may help lower injury rates. Middle‐aged adults may benefit from strength and flexibility training aimed at preserving joint function and preventing overuse injuries. For geriatric climbers, fall‐prevention strategies are critical and should include proprioceptive training, balance exercises, and climbing route modifications to reduce fall height and complexity.^23^, ^24^ Tailoring interventions to the needs of each age group can help promote safety and longevity in the sport.

### Limitations

This study presents several limitations. First, the retrospective design and reliance on the NEISS database limit our ability to capture complete injury details, as a proportion of MOIs reported in this analysis were labeled as “unspecified” (11.0%) due to insufficient narrative detail. Additionally, the NEISS dataset did not have data on climbing frequency, duration of activity, or participant skill level, which would have been useful to inform tailored injury prevention recommendations based on participation patterns, climbing style, or experience level. Third, because the NEISS database only includes emergency department visits, our data may be biased toward higher‐acuity cases, failing to capture injuries that were managed at home, at an urgent care, or by a primary care provider. Consequently, generalization of these results to the broader rock‐climbing population may not accurately depict injury trends. Finally, due to the acute‐care setting of the NEISS data, this study disproportionately represents traumatic events and cannot reliably account for chronic or overuse climbing injuries.

## CONCLUSIONS

In this study, we found that younger climbers experienced more upper extremity injuries, while older adults more commonly sustained lower extremity injuries. In addition, male rock climbers experienced higher proportions of shoulder injuries compared with female rock climbers. These findings illustrate the potential need for age‐ and sex‐specific injury prevention strategies to guide safe participation in this growing sport.

## DISCLOSURES

The authors (T.Z.A., P.M.L., N.N.K., K.B.A., K.K.O., A.J.L., J.M.W.) declare that they have no known competing financial interests or personal relationships that could have appeared to influence the work reported in this paper.
